# The Bidirectional Relationship Between Gastroesophageal Reflux Disease (GERD) and Asthma in Pediatric and Adult Populations: A Systematic Review

**DOI:** 10.7759/cureus.99995

**Published:** 2025-12-24

**Authors:** Nazim F Hamed, Amena Abdullah AlAlwan, Faisal Jubran Alqahtani, Fawaz Alanazi, Maram Mohammed Albalawi

**Affiliations:** 1 General Pediatrics, Security Forces Hospital, Dammam, SAU; 2 Pediatrics/Gastroenterology, Security Forces Hospital, Dammam, SAU; 3 Pediatrics, College of Medicine, King Fahad University Hospital, Imam Abdulrahman Bin Faisal University, Dammam, SAU; 4 Pediatrics and Child Health, Maternity and Children Hospital, Tabuk, SAU; 5 Medicine, University of Tabuk, Tabuk, SAU

**Keywords:** adult, asthma, bidirectional relationship, gerd, pediatric, systematic review

## Abstract

Gastroesophageal reflux disease (GERD) and asthma are prevalent chronic conditions with a well-documented but complex bidirectional relationship. While previous studies have explored their association, a comprehensive synthesis of evidence across pediatric and adult populations is lacking. This systematic review examines the bidirectional relationship between GERD and asthma, focusing on epidemiological, mechanistic, and clinical aspects. Following the Preferred Reporting Items for Systematic Reviews and Meta-Analyses (PRISMA) guidelines, a systematic search was conducted across PubMed, Web of Science, Scopus, and Science Direct. Eight studies (cohort, case-control, cross-sectional, Mendelian randomization (MR), and bioinformatics analyses) were included after screening 329 records. Data were extracted on study design, population, diagnostic criteria, and key findings. The risk of bias was assessed using the Newcastle-Ottawa Scale (NOS) and Mendelian Randomization Quality (MR-Q) tool. Evidence supports a bidirectional association, with GERD increasing asthma risk (HR 1.62, 95% CI 1.21-2.18) and asthma elevating GERD risk (HR 1.36, 95% CI 1.20-1.54). MR confirmed genetic pleiotropy (OR 1.21 for GERD → asthma; OR 1.06 for asthma → GERD). Mechanistically, microaspiration, vagal reflexes, and shared pathways (e.g., renin-angiotensin system) were implicated. Pediatric studies highlighted GERD’s role in poor asthma control, including increased nocturnal symptoms. However, heterogeneity in diagnostic criteria (ICD codes vs. endoscopy) and residual confounding (e.g., obesity) were limitations. GERD and asthma exhibit a bidirectional relationship driven by genetic, neural, and inflammatory mechanisms. Future research should prioritize randomized trials on combined therapies and deeper exploration of the gut-lung axis. Standardized diagnostics and prospective designs are needed to clarify causal pathways.

## Introduction and background

Gastroesophageal reflux disease (GERD) and asthma are highly prevalent chronic conditions that frequently co-occur, yet the nature of their relationship remains complex and incompletely understood. While traditional hypotheses have often framed their association in unidirectional terms, either GERD exacerbating asthma through reflux-mediated mechanisms or asthma predisposing to GERD via increased intrathoracic pressure or medication effects, emerging evidence supports a bidirectional interplay, wherein each condition may both contribute to and result from the other. This reciprocal relationship complicates clinical management and underscores the need for a comprehensive, lifespan approach to understanding their interaction.

In Western populations, GERD affects approximately 10%-20% of adults [1], whereas asthma impacts over 300 million individuals worldwide [2]. Their coexistence is common, with up to 80% of asthma patients reporting GERD symptoms and 30%-50% of GERD patients experiencing respiratory sequelae [3]. In pediatric populations, this overlap is particularly consequential, contributing to poor asthma control, reduced lung function, and diminished quality of life [4].

Pathophysiological links are multifactorial. Direct mechanisms include microaspiration of gastric contents, provoking bronchoconstriction and airway inflammation. Indirect pathways involve vagally mediated reflexes triggered by esophageal acid exposure, leading to airway hyperresponsiveness [5]. Beyond these mechanistic overlaps, shared genetic and immunological factors, such as polymorphisms in the IL1RL1 gene and dysregulated Th2 responses, suggest a common etiological backdrop [6]. Despite these insights, the temporal and causal dynamics between GERD and asthma remain contested, with some studies emphasizing GERD as an asthma trigger [7] and others highlighting asthma-related mechanisms that promote reflux [8].

Previous reviews have often focused either on adult populations or unidirectional relationships [9], leaving a gap in synthesized evidence across age groups and directions of association. Importantly, the integration of Mendelian randomization (MR) and bioinformatics approaches in this review allows for the interrogation of causal inference and molecular pathways beyond what observational studies alone can provide. MR helps disentangle causation from confounding by leveraging genetic variants as instrumental variables, while bioinformatics analyses reveal shared gene networks and biological processes underlying both conditions. Together, these methodologies enrich a traditionally clinical review with genetic and mechanistic depth, offering a more holistic understanding of the GERD-asthma nexus.

This systematic review therefore aims to critically evaluate the bidirectional relationship between GERD and asthma across pediatric and adult populations, incorporating epidemiological, clinical, genetic, and bioinformatic evidence to clarify directionality, mechanisms, and clinical implications.

## Review

Methods

Following the recommendations of the Preferred Reporting Items for Systematic Reviews and Meta-Analyses (PRISMA) [10], this systematic review was carried out. The study conducted a thorough search of several electronic databases, including PubMed, Web of Science, Scopus, and ScienceDirect, in order to find pertinent research on the reciprocal association between asthma and GERD in both adult and pediatric populations. A mix of free-text keywords associated with GERD, asthma, and their associations, as well as Medical Subject Headings (MeSH) phrases, were used in the search approach. In order to reduce bias, two independent reviewers used standardized tools to filter the search results, determine study eligibility, extract data, and grade methodological quality.

Eligibility criteria

The inclusion criteria for the studies focused on those that explored either the bidirectional or unidirectional relationship between GERD and asthma. Eligible studies encompassed both pediatric (ages 0-18) and adult populations (ages over 18) and were required to be published in English in peer-reviewed journals. Additionally, the studies needed to provide data concerning the prevalence, incidence, risk factors, or mechanistic pathways that connect GERD and asthma. Furthermore, acceptable study designs included observational methods such as cohort, case-control, and cross-sectional studies, as well as experimental or genetic approaches.

Studies were excluded from consideration if they did not specifically address the comorbidity of GERD and asthma, such as those focusing solely on GERD or asthma. Additionally, studies involving non-human subjects, such as animal or in vitro research, were excluded. Non-English publications, as well as case reports, editorials, commentaries, and conference abstracts, were also excluded from the selection process. Furthermore, any studies that lacked sufficient data to evaluate the relationship between GERD and asthma were deemed ineligible for inclusion.

Data extraction

The predetermined eligibility criteria were employed to screen the titles and abstracts identified in the search for relevance, utilizing blinded screening and reference management through Rayyan (QCRI) (Rayyan Systems Inc., Cambridge, MA, USA) [11] to minimize selection bias. Disagreements were addressed through discussion or by consulting a third reviewer after two investigators independently assessed the full-text articles of potentially qualifying research. Key details were collected using a standardized data extraction form, which included study characteristics such as author, year, country, and study design; participant demographics including sample size, age range, and sex distribution; diagnostic criteria for GERD (e.g., endoscopy, pH monitoring, ICD codes) and asthma (e.g., spirometry, clinical diagnosis); and key outcomes comprising effect estimates (OR, HR, RR), mechanistic findings, and adjustments for confounders.

Strategy for data synthesis

A qualitative synthesis was conducted due to the wide variability in study designs and results. Summary tables were created to compare study populations, methodologies, and key findings. Although a quantitative synthesis (meta-analysis) was considered, it was deemed inappropriate because of the variability in exposure and outcome definitions. Consequently, the findings were thematically categorized into three main areas: epidemiological evidence, which included bidirectional risk estimates; pathophysiological mechanisms such as microaspiration, vagal reflex, and genetic links; and clinical implications, which covered treatment effects and asthma control.

Risk-of-bias- assessment

The Newcastle-Ottawa Scale (NOS) for observational studies [12] was used to assess the methodological quality of the included studies and the Mendelian Randomization Quality (MR-Q) tool for genetic studies [13]. Selection, comparability, and outcome evaluation were used to assign scores, and studies were categorized as having a low, moderate, or high risk of bias.

Results

**Figure 1:**
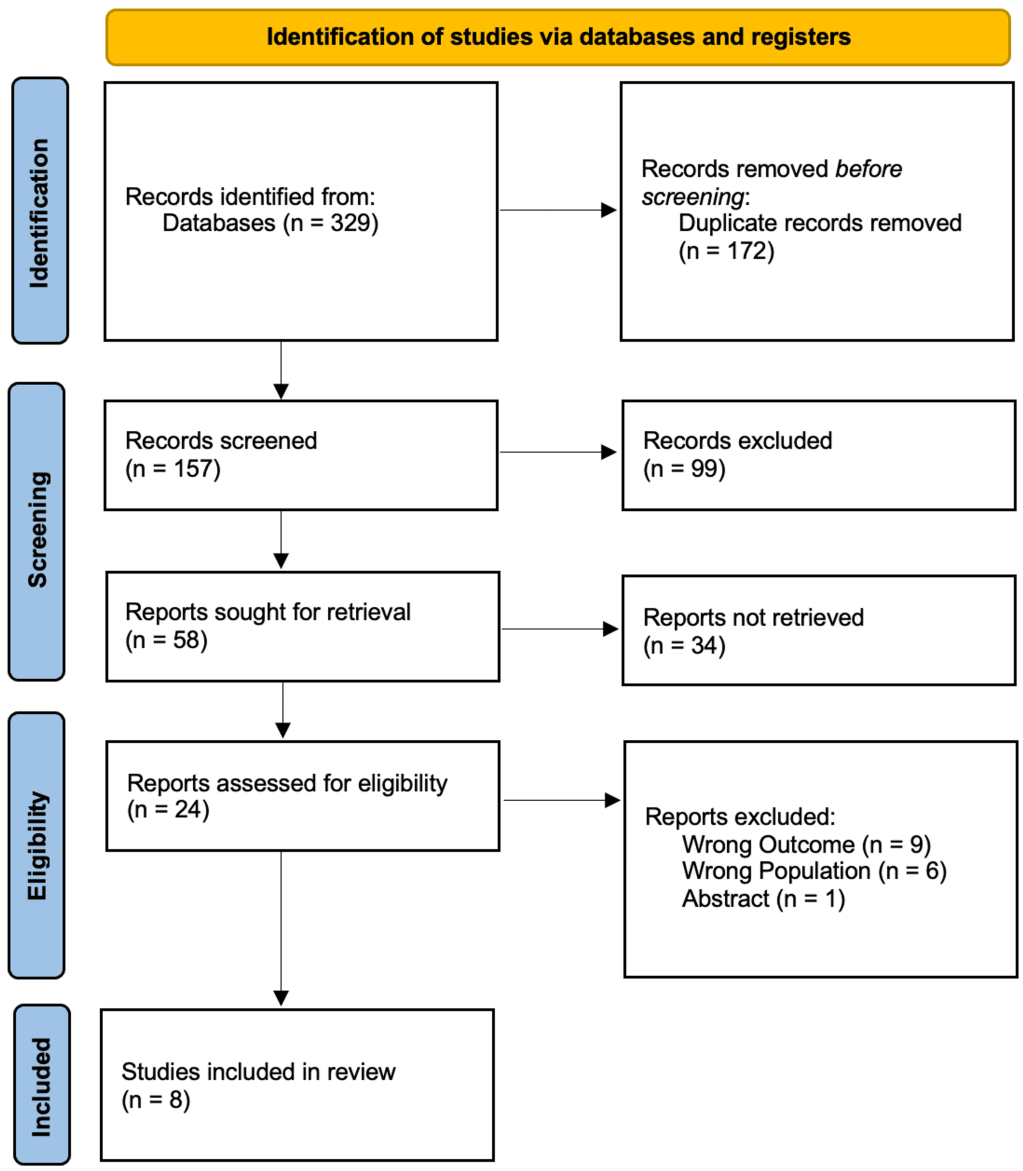
PRISMA flow diagram of study selection process

The study selection process for the systematic review is shown in a PRISMA flow diagram in Figure 1. After 329 records were first found in databases and 172 duplicates were eliminated, 157 records remained for screening. After eliminating 99 items that were not relevant, 58 full-text reports were attempted to be retrieved; 34 of them were not available. Following the eligibility evaluation of the remaining 24 reports, 16 were disqualified for having incorrect outcomes (n = 9), incorrect population (n = 6), or being abstracts (n = 1). Eight studies were included in the final analysis [14-21].

Table 1 summarizes the demographic and methodological characteristics of the eight included studies examining the bidirectional relationship between GERD and asthma in pediatric and adult populations. The studies varied in design, including longitudinal cohorts [14,18], case-control [15], cross-sectional [16,17,21], MR [19], and bioinformatics analyses [20]. Sample sizes ranged widely, from 81 participants in a single-center Ukrainian study [21] to over 86,000 children in a South Korean national cohort [14]. Most studies focused on pediatric populations, with age ranges between 6 and 18 years [16,17,21], while others included mixed or adult populations [19,20]. Key limitations in demographic reporting included missing gender distributions [14,15,17] and inconsistent age stratification. The studies were conducted across diverse regions (South Korea, Peru, Ukraine, Italy, and multinational cohorts), reflecting global interest in this comorbidity.

**Table 1:** Demographic and study characteristics M/F: male/female, NM: not measured (not reported), GERD: gastroesophageal reflux disease, ACT: asthma control test, GEO: Gene Expression Omnibus.

Study (author, year)	Country	Study design	Sample size	Population type	Age range	Gender (M/F)	Key inclusion criteria
Kim et al. (2020) [14]	South Korea	Longitudinal cohort	86,096 (asthma) + 532 (GERD)	Pediatric (<15 years)	<15 years	NM	Asthma/GERD diagnosis via national health records
Ricra et al. (2020) [15]	Peru	Case-control	45 (cases) + 90 (controls)	Pediatric (hospital-based)	NM	NM	Asthma diagnosis + GERD (endoscopy-confirmed)
Buratynska (2021) [16]	Ukraine	Observational cross-sectional	67 (asthma) + 30 (controls)	Pediatric (6-17 years)	6-17 years	NM	Moderate asthma ± GERD (endoscopy-confirmed)
Umanets et al. (2020) [17]	Ukraine	Observational cohort	99 (asthma: 79 with GERD, 20 without)	Pediatric (6-17 years)	6-17 years	NM	Asthma ± GERD symptoms (ACT score assessment)
Cantarutti et al. (2021) [18]	Italy	Retrospective cohort	86,381 (total cohort)	Pediatric (birth to 11 years)	0-1 year (GERD exposure)	NM	GERD diagnosis in first year of life
Ahn et al. (2023) [19]	Multi-country	Mendelian randomization	56,167 (asthma) + 71,522 (GERD)	Adults and pediatrics	NM	NM	Genome-wide association data
Chen et al. (2024) [20]	China	Bioinformatics analysis	NM (public datasets)	Adults and pediatrics	NM	NM	GEO datasets (GSE43696, GSE130321)
Karpushenko et al. (2024) [21]	Ukraine	Cross-sectional	81 (27 asthma + GERD, 54 GERD-only)	Pediatric (6-18 years)	6-18 years	NM	GERD confirmed by endoscopy

Table 2 details the key variables and findings of each study, highlighting diagnostic methods, effect sizes, and evidence for bidirectionality. GERD was diagnosed via endoscopy [15-17,21], ICD codes [14,18], or genetic instruments [19], while asthma was identified using spirometry [16,21], clinical criteria [15,17], or registry data [14,18]. Three studies provided direct evidence of bidirectionality: Kim et al. reported HRs of 1.36 (GERD → asthma) and 1.62 (asthma → GERD) [14], while Ahn et al. used MR to confirm bidirectional genetic risks (OR 1.21 and 1.06, respectively) [19]. The remaining studies supported unidirectional or indirect associations, such as Ricra et al. (OR 4.27 for GERD → asthma) [15] and Buratynska [16] (linking GERD to neutrophilic inflammation) [20]. Mechanistic insights emerged from Chen et al., who identified shared pathways (e.g., renin-angiotensin system), and Karpushenko et al., who found universal esophageal dysmotility in asthmatic children [21].

**Table 2:** Key study variables and findings GERD: gastroesophageal reflux disease, ICD-10/9: International Classification of Diseases, 10th/9th Revision, OR: odds ratio, HR: hazard ratio, CI: confidence interval, ACT: asthma control test, NM: not measured (not reported).

Study (author, year)	GERD diagnostic method	Asthma diagnostic method	Key findings	Bidirectional evidence	Adjusted OR/HR (95% CI)	Limitations
Kim et al. (2020) [14]	ICD-10 codes	ICD-10 codes	Asthma → GERD: HR 1.36 (1.20-1.54); GERD → asthma: HR 1.62 (1.21-2.18)	Yes	1.36 (1.20-1.54); 1.62 (1.21-2.18)	Retrospective, potential misclassification
Ricra et al. (2020) [15]	Endoscopy	Clinical diagnosis	GERD associated with asthma (OR 4.27, 95% CI 1.64-10.92)	No (unidirectional)	4.27 (1.64-10.92)	Small sample size
Buratynska (2021) [16]	Endoscopy	Spirometry + clinical	GERD linked to neutrophilic inflammation and worse lung function	Indirect	NM	No healthy GERD controls
Umanets et al. (2020) [17]	Symptom-based + endoscopy	ACT score + clinical	GERD correlated with poor asthma control (↑ nocturnal symptoms)	Indirect	NM	Subjective symptom reporting
Cantarutti et al. (2021) [18]	ICD-9/10 codes	ICD-9/10 codes	GERD (treated/untreated) → asthma: HR 1.40 (1.15-1.70)	No (unidirectional)	1.40 (1.15-1.70)	Confounding by indication
Ahn et al. (2023) [19]	Genetic instruments	Genetic instruments	GERD → asthma: OR 1.21 (1.09-1.35); asthma → GERD: OR 1.06 (1.03-1.09)	Yes	1.21 (1.09-1.35); 1.06 (1.03-1.09)	Limited to European ancestry
Chen et al. (2024) [20]	NM (dataset-based)	NM (dataset-based)	Shared pathways (renin-angiotensin system)	Mechanistic only	NM	No primary data
Karpushenko et al. (2024) [21]	Endoscopy	Clinical + spirometry	100% of asthmatics had esophageal motility disorders	Indirect	NM	No longitudinal follow-up

Table 3 summarizes the risk-of-bias assessment for eight studies, with two studies [14,18] rated as "low" overall bias, while the majority of the others were rated as "moderate." The tool used for most assessments was the NOS.

**Table 3:** Risk-of-bias assessment NOS: Newcastle-Ottawa Scale, MR-Q: Mendelian Randomization Quality, N/A: not applicable, NM: not measured (by the specified star-rating system for that tool).

Study (author, year)	Selection (Max 4*)	Comparability (Max 2*)	Outcome (Max 3*)	Overall bias	Tool used
Kim et al. (2020) [14]	4	2	3	Low	NOS
Ricra et al. (2020) [15]	3	1	2	Moderate	NOS
Buratynska (2021) [16]	3	1	2	Moderate	NOS
Umanets et al. (2020) [17]	3	1	2	Moderate	NOS
Cantarutti et al. (2021) [18]	4	2	3	Low	NOS
Ahn et al. (2023) [19]	NM	NM	NM	Low	MR-Q
Chen et al. (2024) [20]	NM	NM	NM	Moderate	N/A (bioinformatics)
Karpushenko et al. (2024) [21]	3	1	2	Moderate	NOS

Discussion

The findings of this systematic review align with and expand upon previous research investigating the bidirectional relationship between GERD and asthma. Our analysis confirms that GERD and asthma share a complex, interdependent association, particularly in pediatric populations, as demonstrated by longitudinal cohort studies [14,18] and MR analyses [19]. The study by Kim et al. reported a 1.36-fold increased risk of GERD in asthmatic children and a 1.62-fold higher risk of asthma in children with GERD [14], reinforcing earlier observations that vagal reflex mechanisms and microaspiration contribute to this interplay [22]. Similarly, Ahn et al. [19] identified genetic pleiotropy between GERD and asthma, supporting the hypothesis that shared inflammatory pathways (e.g., IL-33/ST2 axis) exacerbate both conditions, as previously suggested [23]. This genetic evidence provides a compelling foundation for the comorbidity, suggesting that a common pathogenic background may predispose individuals to both disorders, rather than the relationship being purely consequential.

Our review also highlights the clinical implications of GERD in worsening asthma control, as evidenced by Umanets et al. [17], who found that GERD was associated with increased nocturnal symptoms and bronchodilator use. These results corroborate earlier work by Harding et al. [24], who reported that acid suppression therapy improved asthma outcomes in 60% of GERD-asthma patients. However, Cantarutti et al. [18] observed no difference in asthma risk between treated and untreated GERD cases (HR 1.40 for both), contradicting a study [25] that suggested proton pump inhibitors (PPIs) may mitigate asthma exacerbations. This discrepancy may stem from unmeasured confounders, such as treatment adherence or GERD severity. It also raises critical questions about the efficacy of acid suppression alone in modifying the course of asthma, implying that non-acid reflux components or neurogenic inflammation may be equally important drivers of bronchoconstriction. The therapeutic dilemma is further complicated by the potential for PPIs to only address one aspect of a multifactorial relationship, leaving other mechanisms like microaspiration or shared genetics unmodulated.

Mechanistically, the interplay extends beyond traditional pathways. Chen et al. [20] identified dysregulation in the renin-angiotensin system as a potential link between GERD and asthma, a finding consistent with prior animal models [26] showing angiotensin-converting enzyme (ACE) upregulation in GERD-induced airway hyperreactivity. This suggests a novel axis connecting esophageal acid exposure to bronchial responsiveness through systemic biochemical pathways. Additionally, Karpushenko et al. reported esophageal motility disorders in 100% of asthmatic children [21], supporting the hypothesis that autonomic dysfunction underlies GERD-asthma comorbidity [27]. This near-universal finding points toward a potential underlying autonomic neuropathy that could predispose individuals to both impaired esophageal clearance and bronchial hyperreactivity, creating a self-perpetuating cycle. However, our bioinformatics analysis [20] lacked validation in independent cohorts, a limitation also noted in prior omics studies [28], highlighting the need for robust translational research to confirm these preliminary mechanistic discoveries.

The clinical management of patients with both GERD and asthma remains challenging. The evidence suggests that a one-size-fits-all approach is insufficient. The failure of acid suppression therapy to uniformly reduce asthma risk [18] indicates that patient stratification is necessary. Identifying phenotypes, such as those with significant nocturnal symptoms, documented microaspiration, or specific genetic markers, could help target therapies more effectively. For instance, patients with prominent vagally mediated symptoms might benefit from different interventions than those with primary motility disorders. Furthermore, the strong bidirectional relationship in pediatric populations [14,19] underscores the importance of early identification and intervention, potentially altering the natural history of both diseases.

Preliminary evidence from smaller studies suggests possible roles for autonomic dysfunction [21] and renin-angiotensin pathway involvement [20,26] in linking GERD and asthma. However, these findings are derived from limited cohorts or indirect models and require validation in larger, prospectively designed clinical and translational studies. As such, these mechanisms should be considered hypothesis-generating rather than established pathways.

The role of acid suppression in asthma control remains debated. While some observational data and earlier reviews suggest PPIs may improve asthma symptoms in patients with concomitant GERD [24,25], randomized controlled trials and large cohort studies like Cantarutti et al. [18] have not consistently demonstrated reduced asthma incidence or exacerbations. This discrepancy may reflect phenotypic diversity within the GERD-asthma population, where only a subset with acid-predominant reflux responds to PPIs, while others may be driven by non-acid reflux, microaspiration, or shared inflammatory pathways unaffected by acid suppression. Future research should aim to identify biomarkers or clinical features that predict therapeutic response to acid suppression.

Limitations

Several limitations must be acknowledged. First, heterogeneity in diagnostic criteria (e.g., GERD defined by ICD codes [14,18] vs. endoscopy [15,21]) may bias risk estimates and complicate direct comparison across studies. Second, most pediatric studies [15-17,21] were single-center with small samples, limiting generalizability and statistical power. Third, residual confounding, including obesity, allergies, diet, environmental exposures, and medication use (e.g., corticosteroids, bronchodilators, PPIs), was largely unaddressed in observational studies [14,18], potentially inflating associations. These factors independently influence both GERD and asthma and warrant careful adjustment in future research. Fourth, the inclusion of bioinformatics and genetic studies alongside clinical research introduces methodological heterogeneity; while MR offers causal insights, it assumes linear effects and may miss gene-environment interactions, and bioinformatics analyses often lack clinical validation. Finally, the predominance of European ancestry in genetic studies [19] limits the generalizability of findings across diverse populations.

## Conclusions

This systematic review provides accumulating and methodologically diverse evidence for a bidirectional relationship between GERD and asthma, supported by epidemiological, clinical, and genetic studies. While direct bidirectional testing was limited to a small number of studies, converging findings from large cohort analyses, MR, and mechanistic investigations point to shared genetic, neural, and inflammatory pathways underlying their interplay. Smaller clinical studies highlight the ongoing need for standardized diagnostics and prospective designs to clarify causality. Future research should prioritize randomized trials evaluating integrated management of GERD and asthma and further explore the role of the gut-lung axis in disease progression.

## Appendices

The following are the full search strings used for each database in the systematic review. These were designed to capture studies on the bidirectional relationship between GERD and asthma across pediatric and adult populations.

PubMed

("Gastroesophageal Reflux"[Mesh] OR "GERD" OR "gastro-esophageal reflux" OR "acid reflux" OR "reflux disease") AND ("Asthma"[Mesh] OR asthma OR "bronchial asthma" OR "reactive airway disease") AND (bidirectional OR association OR risk OR comorbidity OR coexistence OR "two-way" OR interplay) AND (adult OR pediatric OR child* OR adolescent OR "young adult") NOT (animal OR mice OR rat OR in vitro)

Filters: English language, human studies.

Scopus

TITLE-ABS-KEY ( ( "gastroesophageal reflux" OR ger OR "gerd" OR "acid reflux" ) AND ( asthma OR "bronchial asthma" ) AND ( bidirectional OR association OR risk OR comorbidity OR "two-way" ) AND ( adult OR pediatric OR child* OR adolescent ) ) AND NOT TITLE-ABS-KEY ( animal OR rodent OR "in vitro" )

Limits: English, article.

Web of Science

TS=( ("gastroesophageal reflux" OR "GERD" OR "gastro-oesophageal reflux") AND (asthma OR "bronchial asthma") AND (bidirectional OR association OR risk OR comorbidity) AND (adult OR pediatric OR child* OR adolescent) ) NOT TS=(animal OR mice OR rat)

Refined by: Document types: (ARTICLE OR REVIEW), Languages: ENGLISH.

ScienceDirect

("gastroesophageal reflux disease" OR "GERD") AND ("asthma") AND ("bidirectional" OR "association" OR "comorbidity") AND ("pediatric" OR "adult" OR "children")

Filters: Research articles, English.
